# Development and external validation of machine-learning models for diagnosing malignant pleural effusion

**DOI:** 10.3389/fonc.2026.1880122

**Published:** 2026-08-17

**Authors:** Aihua Wu, Shanshan Wang, Hongying Ma, Songbo Yuan, Yanqing Liu

**Affiliations:** 1 Department of Laboratory Medicine, The First Affiliated Hospital of Ningbo University, Ningbo, Zhejiang, China; 2 Department of Respiratory and Critical Care Medicine, The First Affiliated Hospital of Ningbo University, Ningbo, Zhejiang, China; 3 Department of Laboratory Medicine, The Affiliated People’s Hospital of Ningbo University, Ningbo, Zhejiang, China; 4 School of Medicine, Ningbo University, Ningbo, China

**Keywords:** area under the curve, diagnostic performance, extreme gradient boosting, machine learning, malignant pleural effusion

## Abstract

**Background:**

Clinicians face challenges in diagnosing malignant pleural effusion (MPE) and distinguishing it from benign causes. This study aimed to develop and validate machine learning (ML) models for this purpose.

**Methods:**

The retrospective study included 1,530 untreated patients with pleural effusion (PE) between January 2016 and December 2025. Clinical variables, including age, sex, smoking status, and laboratory indices were collected for analysis. The patients were randomly divided into the training and test sets at a ratio of 7:3. Six ML algorithms were developed and compared to determine the best diagnostic model for MPE using seven metrics, calibration curve, and decision curve. The SHapley Additive exPlanations (SHAP) values were used to interpret the model. An independent cohort of 244 PE patients was used for external validation.

**Results:**

Based on the feature importance analysis, the XGBoost (extreme gradient boosting) model achieved the best diagnostic performance in the training set, with an AUC of 0.988 (95% CI: 0.982-0.993), the lowest brier score 0.037 (95% CI: 0.029-0.045), and high values for accuracy of 0.951, sensitivity of 0.891, specificity of 0.984, and F1 score of 0.928. Six features were included: fluid carcinoembryonic antigen (CEA), serum CEA, serum cytokeratin 19 fragment (CYFRA 21-1), fluid carbohydrate antigen 724 (CA724), fluid carbohydrate antigen CA199 (CA199), and fluid adenosine deaminase (ADA). SHAP analysis showed that fluid CEA, fluid ADA, and serum CYFRA21–1 were the three most important variables.

**Conclusions:**

We developed and validated an accurate and interpretable XGBoost model for distinguishing MPE from BPE using routine laboratory data, which might be applied in clinical practice and decision-making.

## Introduction

Pleural effusion (PE) is a common clinical problem, with more than 50 possible etiologies (1). Although over 75% of PE cases originate from malignancy, tuberculosis, pneumonia, and heart failure, the diagnosis of PE is still challenging in clinical settings (1–3). Evidence shows that approximately 20% of PE cases remain undiagnosed (1). Among these, malignant pleural effusion (MPE) is a life-threatening complication, caused by various cancer, including both primary pleural malignancy (mesothelioma) and the result of secondary spread from other sites (lung, breast, bone marrow, and ovarian) (2). It is estimated that approximately 500,000 new MPE cases occur in the USA and Europe (2, 4). In China, the incidences of lung cancer, breast cancer, and hematologic tumors rank first globally. Evidence shows that MPE accounts for 23.7% of all causes of PE in China (5). Given the short median survival for MPE (3–12 months), appropriate oncologic and palliative management should be initiated as soon as possible upon a patient being diagnosed with MPE. Therefore, early diagnosis and differential diagnosis from other types of PE are critical to the prognosis and survival of MPE patients.

Cytologic examination of pleural fluid for diagnosing MPE is a routine method in the clinic. The sensitivity of PE cytology for diagnosing adenocarcinomas can reach over 70%, however, its sensitivities for other tumor types are lower than 50% (6). At present, closed pleural biopsy and thoracentesis have become routine procedures to clarify the cause of unknown PE. Closed pleural biopsy has a lower diagnostic sensitivity than cytology, nevertheless, its sensitivity can reach over 85% when used with computed tomography (CT) or ultrasound guidance (1, 7). Even with newer needles (Tru-Cut, Biopty TM, and Temno) and imaging guidance, the sensitivity of closed pleural biopsy for diagnosing MPE remains lower than that of thoracoscopy. Hence, the application of closed pleural biopsy is not preferred for MPE diagnosis when thoracoscopy is available. Besides, thoracentesis is an invasive sampling procedure associated with potential complications, although the operation is ultrasound-guided. However, multiple complications (including pneumothorax, pain, shortness of breath, and hematoma) and moderate diagnostic accuracy (40%-87%) also limit its use (7, 8). Medical thoracoscopy (MT) is a safe and effective diagnostic method for MPE patients with undiagnosed exudates or suspicious pleural abnormalities under imaging examination (9). The sensitivity exceeded 90% for diagnosing MPE using MT, which was not affected by histological type, including mesothelioma (9). Nevertheless, the minimally invasive procedure still has its limitations. The method is strictly contraindicated in patients with severe respiratory distress and whose lungs are adherent to the chest wall throughout the hemithorax. Additionally, several complications, such as infection, bleeding, pneumothorax, bronchopleural fistula formation, periprocedural hypotension, and subcutaneous emphysema are still concerns (7, 9). Under the circumstances, pleural-fluid analysis is a routinely available, less-invasive diagnostic approach. The biochemical markers [including total protein (TP), adenosine deaminase (ADA), and lactate dehydrogenase (LDH)] and tumor markers [including carcinoembryonic antigen (CEA), carbohydrate antigen 15-3 (CA15-3), and carbohydrate antigen 125 (CA125)] could be used for initially distinguishing MPE from benign pleural effusion (BPE). Unfortunately, their low sensitivity, indeterminate cut-off values in PE, and inability to distinguish primary tumor sites also limit their utility in clinical settings.

Machine learning (ML) has been applied to various fields of medicine, such as disease diagnosis, prediction, patient treatment, and drug discovery (10–12). Previous studies have reported the application of ML models in identifying the types of PE, including MPE and tuberculous pleural effusion (TPE) (13–15). Two studies suggested that support vector machine (SVM) is the best model for diagnosing TPE, with an accuracy of more than 95% for both (13, 14). However, Ren et al. showed that the random forest (RF) model performed best in diagnosing TPE, with an area under the curve (AUC) of 0.965 and accuracy of 91.5% (15). However, few studies have explored the application of ML in identifying MPE using clinical and routine laboratory variables. Li et al. showed that the deep-learning model had the highest AUC of 0.995 compared to the other five ML models, namely gradient boosting machine (GBM), extreme gradient boosting (XGBoost), extremely randomized trees (XRT), distributed random forest (DRF), and generalized linear models (GLM) (16). However, only CEA in PE was included in the study. Zhang et al. found that XGBoost exhibited better diagnostic performance for MPE using a single tumor marker and/or combinations of tumor markers (17). Nevertheless, the small size (only 391 cases), lack of tumor markers in the blood, and limited set of performance metrics might compromise the results.

In the present study, we developed six ML models based on clinical and laboratory variables to distinguish MPE from BPE. Seven metrics were used to compare and identify the best-performing model, such as accuracy, sensitivity, specificity, and AUC. Besides, Shapley additive explanations (SHAP) analysis was used to interpret the optimal model and key features. Finally, independently external cohorts were used to validate our results.

## Materials and methods

### Study design and patient selection

A cohort of 1,906 patients with all-caused PE was admitted to the First Affiliated Hospital of Ningbo University between January 2016 and December 2025. Patients were included under the following criteria: (a) PE patients underwent examination by either ultrasonography, X-ray, or chest CT; (b) PE patients underwent examination by either cytology, pleural biopsy, or thoracentesis; (c) PE patients were followed up ≥ 6 months. The exclusion criteria were: (a) patients who underwent antituberculosis therapy before admission; (b) age < 18 years old and/or pregnant women; (c) patients with incomplete clinical and laboratory data of more than 20%; (d) PE patients with any two malignancies or severe lung disease; (e) unknown etiologies of PE. All PE patients included in the study were newly diagnosed and untreated anywhere before. MPE was defined as pleural effusion caused by malignancy, confirmed by positive pleural fluid cytology or pleural biopsy, or by imaging evidence of malignant pleural nodules or thickening in patients with a known extra-pleural primary tumor. Cytology-negative patients were further investigated with thoracoscopy and/or computed tomography-guided biopsy. BPE was defined as pleural effusion without evidence of malignancy, TPE, parapneumonic pleural effusion, and other pleural effusions with a confirmed benign etiology. Detailed diagnostic criteria for MPE and BPE were shown in **Supplementary Table 1**. The baseline demographic, laboratory indexes, and clinical information of all patients were collected from the clinical electronic record system. Finally, a total of untreated 1,530 PE patients were included. 550 were MPE and the remaining were BPE patients, including 449 TPE patients. An independent cohort of 244 patients with PE collected from the Affiliated People’s Hospital of Ningbo University between March 2021 and September 2025 was used for external validation.

### Data collection

Demographic indexes (sex and age), biochemical markers (such as ADA and LDH), and tumor markers [such as CEA, CA125, and carbohydrate antigen 19-9 (CA19-9)] in blood and PE were collected from the electronic medical record system. All the collected indexes were shown in **Supplementary Table 2**. The measurements of TP, glucose, and high-sensitivity C-reactive protein (CRP) were conventional biochemical detection methods, including enzyme method, endpoint method, and immunoturbidimetry (concentration). The detections for ADA and LDH were the velocity method (activity). Blood routine counts were performed using Sysmex XN-9000 automatic hematology analyzer (Tokyo, Japan). Biochemical routine indexes (TP, glucose, ADA, and LDH) were detected using Beckman AU5800 automatic biochemical analyzer (Indiana, USA). The detections for CEA, CA125, and CA19–9 were using chemiluminescence method (Beckman Coulter DXI800, Indiana, USA). The detections for cytokeratin 19 fragment (CYFRA 21-1), neuron-specific enolase (NSE), and carbohydrate antigen 724 (CA724) were using electrochemical luminescence method (Mindray CL-6000, Shenzhen, China).

### Data preprocessing and feature selection

**Figure 1:**
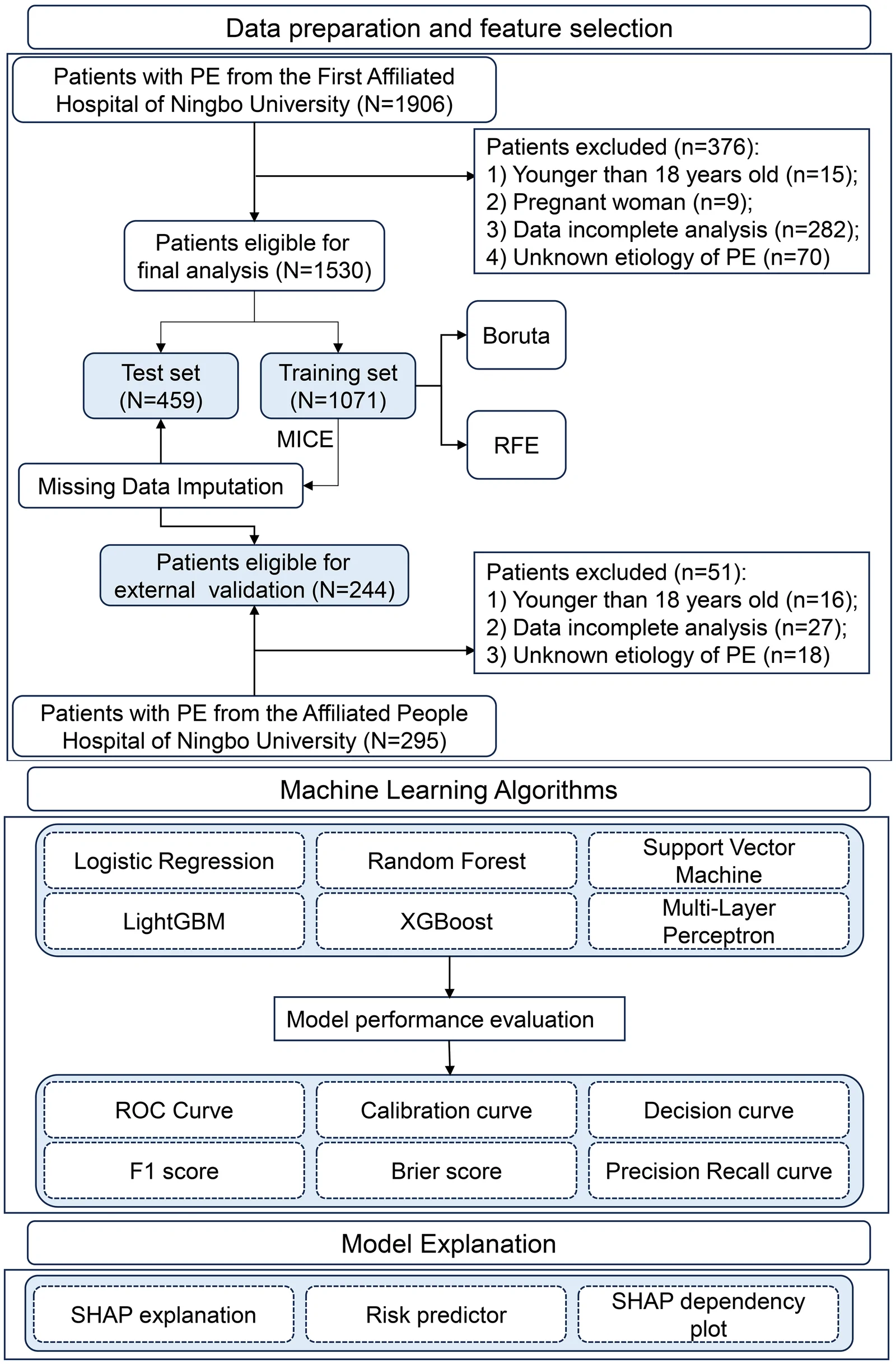
Workflow of model development and validation. The diagram illustrates of the study population, data preprocessing, feature selection using Boruta and recursive feature elimination (RFE), model development and validation using six machine learning algorithms, and performance evaluation with SHAP-based interpretability visualization.

The data preprocessing and model construction workflow was presented in **Figure 1**. The dataset in the training set is divided in a ratio of 7: 3, with 70% used for model training and the remaining 30% are used to test model performance. The collected data contained missing values. Removing all incomplete data might inevitably reduce the sample sizes and the quality of the data, as well as compromise the predictive accuracy. As described above, variables with more than 20% missing data were excluded. The remaining missing values were imputed using multivariate imputation by chained equations (MICE) with 10 iterations and a BayesianRidge estimator. Spearman’s rank correlation coefficients were used to assess correlations among continuous variables. Variance inflation factors (VIFs) were calculated to evaluate multicollinearity among the selected predictors, and variables with VIF >5 were excluded. Boruta, recursive feature elimination (RFE) methods combined with cross-validation were performed for candidate variables selection. Boruta is a wrapper-based feature selection method that aims to identify all features carrying predictive information for the target variable, rather than just the smallest subset that yields optimal model performance. The Boruta algorithm compares the importance of original features with that of shadow features to determine the true importance of each feature. The core parameter settings were as follows: max_iter=50, n_estimators=‘auto’, verbose=2. RFE is a feature selection method that iteratively removes the least important features to identify the most informative subset of predictors. By ranking features according to model-derived importance, RFE helps reduce dimensionality, improve model interpretability, and mitigate overfitting. The refined optimal features were subsequently integrated into the six machine learning models for training and testing.

### Application of ML algorithms

In the study, six distinct ML algorithms were applied to distinguish MPE from BPE, including LR, RF, SVM, MLP, XGBoost and LightGBM. LR is used to generalize linear relationships analysis and is widely used in sociology, clinical medicine, economic forecasting, and quantitative psychology (18). RF, an ensemble classification algorithm, is performed by constructing a multitude of decision trees during the training phase and outputs the mode of the classes (classification) or the mean prediction of the individual trees (19). SVM adopts linear kernel functions and regularization parameters to find an optimal hyperplane in high-dimensional space (19). MLP is a feedforward neural network capable of modeling complex nonlinear relationships for a variety of tasks, including classification, regression, and feature extraction (20). LightGBM, a variant of GB, adopts the strategy of leafwise tree growth that identifies the “best” leaf with the highest gain and only splits the best leaf, resulting in an asymmetrical tree (21). XGBoost is an enhanced implementation of the GB-supervised ML method, which shares similarities with RF but utilizes a more regularized model formulation to reduce data overfitting (21). The detailed explanation of the hyperparameter combination and the determined optimal combination of six ML classifiers were elucidated in the **Supplementary Method**. To enhance classifier performance and mitigate overfitting, we used five-fold cross-validation combined with grid search for hyperparameter optimization within the training set.

### Model performance evaluation and SHAP interpretation

Model performance was rigorously evaluated on both the training set and validation sets. Assessment metrics including AUC, accuracy, sensitivity, specificity, precision, F1 score and brier score. Calibration curves and the brier score assessed the agreement between predicted probabilities and observed probabilities, reflecting model calibration. DCA was performed to estimate net benefit across a range of threshold probabilities, evaluating clinical utility. To enhance model interpretability, we applied SHAP to quantify the contribution of each feature to the best-performing model’s predictive output. Individual and mean SHAP values were calculated, and beeswarm and mean SHAP plots were generated to assess feature importance.

### Statistical analysis

All the statistical analyses were performed by Python (version 3.14.3), scikit-learn (version 1.8.0) and R (version 4.4.1). A two-sided *P* < 0.05 was considered statistical significance. Continuous variables were presented as median and interquartile range (IQR, 25^th^–75^th^), which were compared using the non-parametric Mann–Whitney *U* test. Categorical variables were presented as numbers and percentages (n, %), which were compared using the Chi-square (χ^2^) test or Fisher’s exact test.

## Results

### Patient cohorts and baseline characteristics

Based on inclusion and exclusion criteria, a total of 1,530 newly diagnosed patients with PE were eventually included in the study (**Figure 1**). Patients were randomly assigned to the training cohort (n=1071) and the internal test cohort (n=459) in a 7:3 ratio. The detailed etiologies of MPE and BPE were presented in **Supplementary Table 3**. Among MPE cases, lung cancer was the most common etiology, while TPE accounted for the largest proportion among BPE cases. Baseline demographic and laboratory variables in the training sets are summarized in **Table 1**. Notably, significant statistical differences were observed in age, sex, and most laboratory variables between MPE and BPE groups. Spearman correlation analysis was conducted separately in the MPE and BPE groups to examine relationships among variables and assess potential multicollinearity. The corresponding correlation heatmaps are shown in **Supplementary Figures 1A, B**. In the MPE group, most fluid and serum tumor biomarker demonstrated positive correlation.

**Table 1:** Comparisons of demographic and laboratory variables as well as the corresponding ratios between MPE and BPE patients in the training sets.

Variables	MPE (n = 385)	BPE (n = 686)	*P* value
Age (years) ^a^	70 (62-79)	59 (40-74)	< 0.001
Sex (n, %) ^b^			
Female	150 (41%)	238 (33%)	
Male	235 (59%)	448 (67%)	0.17
Smoking history (n, %) ^b^			
Non-smokers	207 (59%)	439 (63%)	
C/F smokers	178 (41%)	247 (37%)	0.001
Fluid ^a^			
WBC (×10^8^/L)	12.6 (6-23.2)	19.3 (8-39.7)	< 0.001
Neutrophil (×10^8^/L)	1.3 (0.5-3.7)	1.8 (0.6-6.5)	< 0.001
Lymphocyte (×10^8^/L)	7.4 (3.5-13.5)	10.9 (3.7-24.7)	< 0.001
Total protein (g/L)	45.6 (39.6-51.2)	48.5 (38.8-53.1)	0.13
GLU (mmol/L)	6.7 (5.3-8)	5.8 (4.3-7.5)	< 0.001
ADA (U/L)	8.5 (6.2-12.7)	26.4 (9.9-43.2)	< 0.001
LDH (U/L)	362 (227-622)	380.5 (199.5-703.3)	0.82
CEA (ng/mL)	70.6 (7-720.2)	1 (0.7-1.6)	< 0.001
CA125 (U/mL)	1880 (1056-3275)	1000 (433-1833)	< 0.001
CA19-9 (U/mL)	13.5 (2.7-222.8)	2.2 (1.1-4.1)	< 0.001
CYFRA21-1 (ng/mL)	69.9 (30-263.6)	23.7 (11.2-44.3)	< 0.001
NSE (ng/mL)	13.3 (5.7-36.6)	6.8 (3.5-13.6)	< 0.001
CA724 (U/mL)	6.2 (1.3-48.6)	0.9 (0.7-1.3)	< 0.001
Serum ^a^			
CRP (mg/L)	11.9 (3.1-30.2)	35.95 (9.7-84.5)	< 0.001
ESR (mm/h)	33 (17-53)	47 (26-68)	< 0.001
LDH (U/L)	200 (166.5-258)	185.5 (159.8-229.3)	< 0.001
CEA (ng/mL)	9 (3.3-45.62)	1.6 (1-2.4)	< 0.001
CA125 (U/mL)	104.9 (49.3-198.7)	88.9 (44.1-167.7)	0.002
CA19-9 (U/mL)	12 (5.4-41.3)	7 (3.5-12.5)	< 0.001
CYFRA21-1 (ng/mL)	5.3 (3.3-10.4)	1.79 (1.2-2.7)	< 0.001
NSE (ng/mL)	14.9 (12.1-21.8)	11.6 (9.3-14.8)	< 0.001
CA724 (U/mL)	1.9 (1.1-5.6)	1.1 (0.8-2.2)	< 0.001

MPE, malignant pleural effusion; BPE, benign pleural effusion; C/F, current/former; WBC, white blood cell; GLU, Glucose; ADA, adenosine deaminase; LDH, lactate dehydrogenase; CEA, carcinoembryonic antigen; CA125, carbohydrate antigen 125; CA19-9, carbohydrate antigen 19-9; CYFRA21-1, cytokeratin 19 fragment; NSE, neuron-specific enolase; CA724, carbohydrate antigen 724; CRP, high-sensitivity C-reactive protein; ESR, erythrocyte sedimentation rate.

Continuous variables were shown as median and interquartile range (IQR, 25^th^-75^th^). Categorical variables were shown as numbers and percentage (n, %).

^a^Age and other continuous variables between MPE and BPE were compared using Mann-Whitney *U* test.

^b^Sex and smoking history between MPE and BPE were compared using Chi-squared (χ^2^) test.

### Feature selection and variable importance ranking

**Figure 2:**
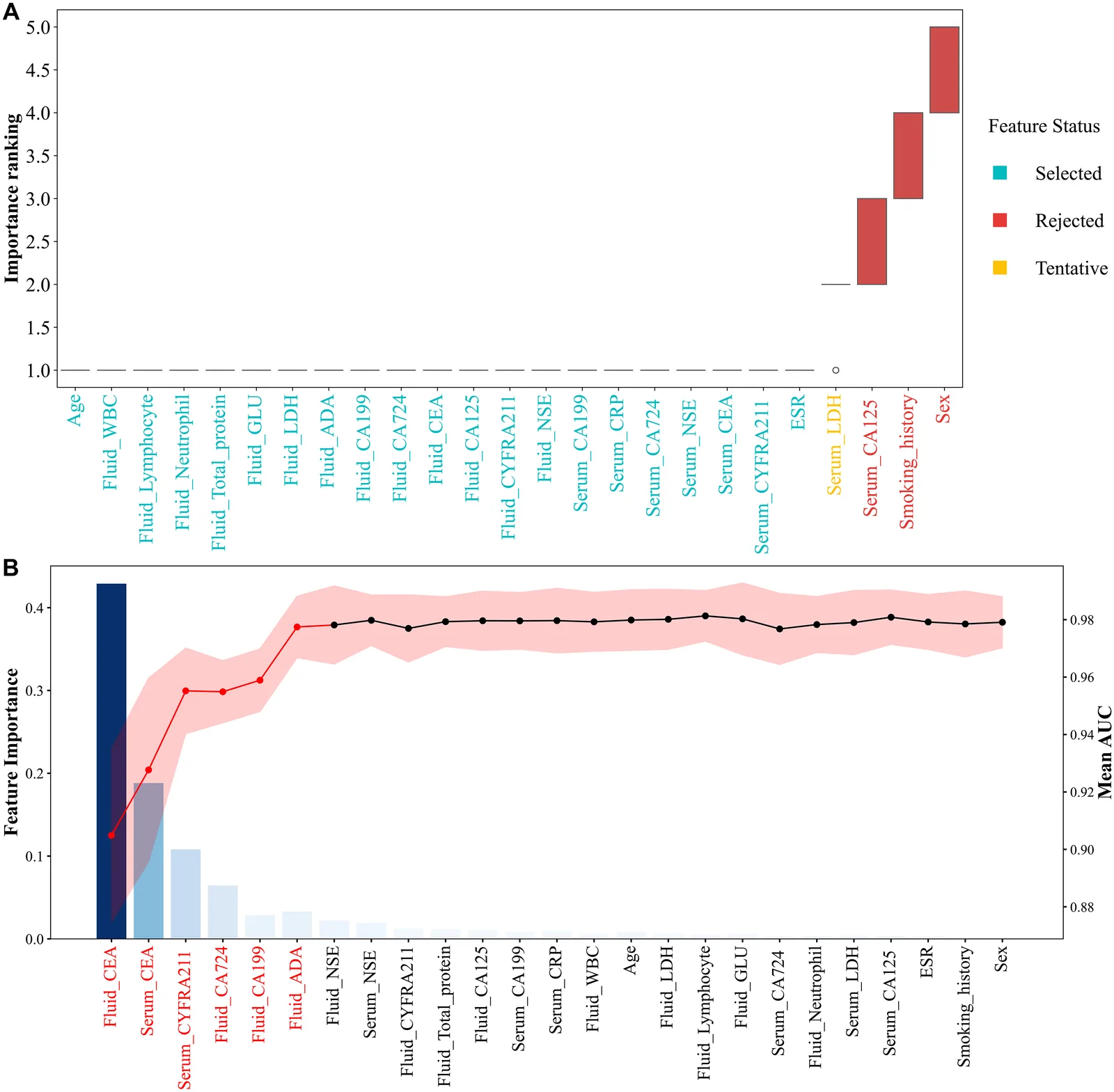
Feature selection process and final predictor selection. **(A)** Feature importance identified by the Boruta algorithm (green: confirmed, red: rejected, yellow: tentative). **(B)** RFE was applied for feature selection. Feature contribution and AUC performance derived from the random forest (RF) model constructed with selected variables were evaluated using five-fold cross-validation.

A total of 25 candidate predictors were collected, including demographic, biochemical markers, and tumor markers from fluid and serum. We employ a wrapper-based feature selection method based on random forests to screen the most relevant predictors. First, the Boruta algorithm, which quantitatively estimates the importance of all relevant features, screened candidate variables and retained 21 features (**Figure 2A**). Next, RFE strategy was applied to avoiding overfitting and reduces the number of features. This recursive feature elimination method is beneficial in improving the performance of the predictive model. According to the 5-fold ROC and feature importance ranking (**Supplementary Table 4**), six predictors were finally selected: Fluid CEA, Serum CEA, Serum CYFRA211, Fluid CA724, Fluid CA199 and Fluid ADA (**Figure 2B**). Additionally, the VIF analysis was performed to assess the collinearity among the selected features. All VIFs were < 2, suggesting no evidence of multicollinearity (**Supplementary Table 5**). The comparisons of the selected six variables for developing machine learning across all cohorts were presented in **Table 2**. The training and the validation sets had similar distributions.

**Table 2:** Comparisons of selected features for developing machine learning in the training, test, and external validation sets.

Features	Training set (n = 1071)		Test set (n= 459)		External validation set (n= 244)	
	MPE (n = 385) BPE (n = 686)		MPE (n = 165) BPE (n = 294)		MPE (n = 115) BPE (n = 129)	
Fluid						
ADA (U/L)	8.5 (6.2-12.6)	26.4 (9.9-42.9)	8.8 (6.1-13.3)	24 (8.8-42.3)	9(6-12)	32 (11-50)
CEA (ng/mL)	69.2 (7.0-789.4)	1 (0.7-1.6)	93.1 (9.2-624.2)	1 (0.7-1.6)	46.1 (9.1-444.3)	1.2 (0.7-1.9)
CA19-9 (U/mL)	13.3 (2.7-212.4)	2.2 (1.1-4.1)	19.3 (3.3-268)	2 (1.2-3.5)	15.5 (2.6-388.2)	2.1 (0.9-4.9)
CA724 (U/mL)	6.2 (1.3-47.9)	1.3 (0.7-3.1)	5.4 (1.4-38.7)	0.9 (0.7-1.3)	3.3 (1.7-19.4)	0.9 (0.8-1.2)
Serum						
CEA (ng/mL)	8.8 (3.3-44)	1.5 (1-2.4)	5.6 (2.8-32.4)	1.5 (1-2.4)	7.5 (2.5-57.8)	1.8 (1.1-2.5)
CYFRA21-1 (ng/mL)	5.3 (3.3-10.3)	1.8 (1.2-2.7)	5.2 (3.5-11.6)	1.8 (1.2-2.8)	6.2 (3.5-13.3)	1.8 (1.5-2.8)

MPE, malignant pleural effusion; BPE, benign pleural effusion; ADA, adenosine deaminase; CEA, carcinoembryonic antigen; CA19-9, carbohydrate antigen 19-9; CA724, carbohydrate antigen 724; CYFRA21-1, cytokeratin 19 fragment.

Continuous variables were shown as median and interquartile range (IQR, 25^th^-75^th^).

### Multiple ML model performance

**Figure 3:**
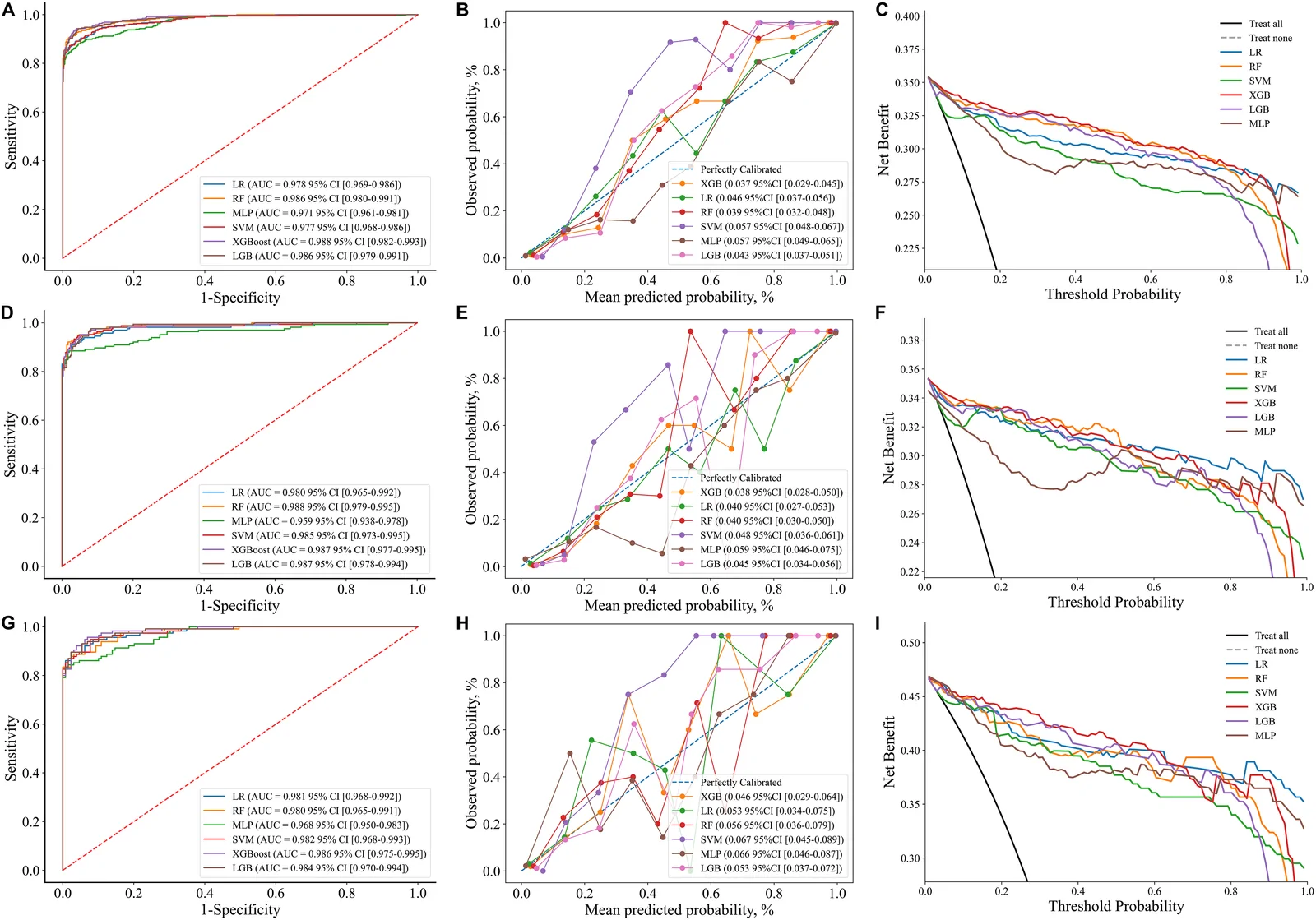
Comparison of model performance in the training, test and validation sets. **(A, D, G)** Receiver operating characteristic (ROC) curves illustrating the discriminative performance of six algorithms in the training, test and the external validation sets. **(B, E, H)** Calibration curves demonstrating agreement between predicted and observed probabilities for each model in the training, test and external validation sets. **(C, F, I)** Decision curve analysis (DCA) evaluating the net clinical benefit of six algorithms across a range of threshold probabilities in the training, test and external validation sets.

We based our model training and testing on the aforementioned selected features. Five-fold cross-validation was conducted in the training cohort for the six machine learning models, and the prediction performance of each model was obtained. The best parameters for each model were presented in supplement method. The AUC of all models on the training, test and validation sets ranged between 0.959 and 0.988 (**Figures 3A, D, G**). Common performance metrics across different ML models in all cohorts are summarized in **Table 3**. In the training set, the XGBoost model achieved the best performance, with an AUC of 0.988 (95% CI: 0.982-0.993), the lowest brier score 0.037 (95% CI: 0.029-0.045), and high values for accuracy (0.951), sensitivity (0.891), specificity (0.984) and F1 score (0.928) (**Figure 3B**). Moreover, the performance of the XGBoost were consistent between the training, test and validation sets, indicating high model robustness (**Figures 3E, H**). DCA curve demonstrated that the XGBoost model yielded a higher net clinical benefit across a range of threshold probabilities, indicating better clinical utility (**Figures 3C, F, I**). The precision-recall (PR) curve (average precision [AP] = 0.98) further validated the model’s precision and robust generalizability (**Supplementary Figures 2A–C**). Overall, the XGBoost model demonstrated the best performance in terms of discrimination, calibration, and clinical utility, and was therefore selected for the final predictive model.

**Table 3:** Performance of six machine learning models in the training, test, and external validation sets.

Datasets	Model	Accuracy (%)	Sensitivity (%)	Specificity (%)	Precision (%)	F1 Score (%)	Brier score (%)	AUC (%)
Training	LR	93.7	85.2	98.4	96.8	90.6	4.6	97.8
	RF	95.3	88.6	99.1	98.3	93.2	3.9	98.6
	MLP	93	85.7	97.1	94.3	89.8	5.7	97.1
	SVM	92.3	79	99.7	99.4	88	5.7	97.7
	XGBoost	95.1	89.1	98.4	96.9	92.8	3.7	98.8
	LGB	94.7	87.5	98.8	97.7	92.3	4.3	98.6
Test	LR	94.8	89.1	98	96.1	92.5	4	98
	RF	95.9	90.9	98.6	97.4	94	4	98.8
	MLP	93.9	88.5	96.9	94.2	91.3	5.9	95.9
	SVM	93.3	81.8	99.7	99.3	89.7	4.8	98.5
	XGBoost	94.8	89.7	97.6	95.5	92.5	3.8	98.7
	LGB	93.9	87.9	97.3	94.7	91.2	4.5	98.7
Validation	LR	89.5	86.1	93.8	94.7	90.2	5.3	98.1
	RF	93.4	88.7	97.7	97.2	92.7	5.6	98
	MLP	89.3	83.5	94.6	93.2	88.1	6.6	96.8
	SVM	89.9	78.5	99.5	99.3	87.7	6.7	98.2
	XGBoost	92.5	88.4	95.6	93.8	91	4.6	98.6
	LGB	90.1	86.5	93.5	92.5	89.4	5.3	98.4

AUC, area under the curve; LR, logistic regression; RF, random forest; MLP, multi-layer perceptron; SVM, support vector machine; XGBoost, extreme gradient boosting; LGB, light gradient boosting machine; AUC, area under the curve.

### Model interpretability

**Figure 4:**
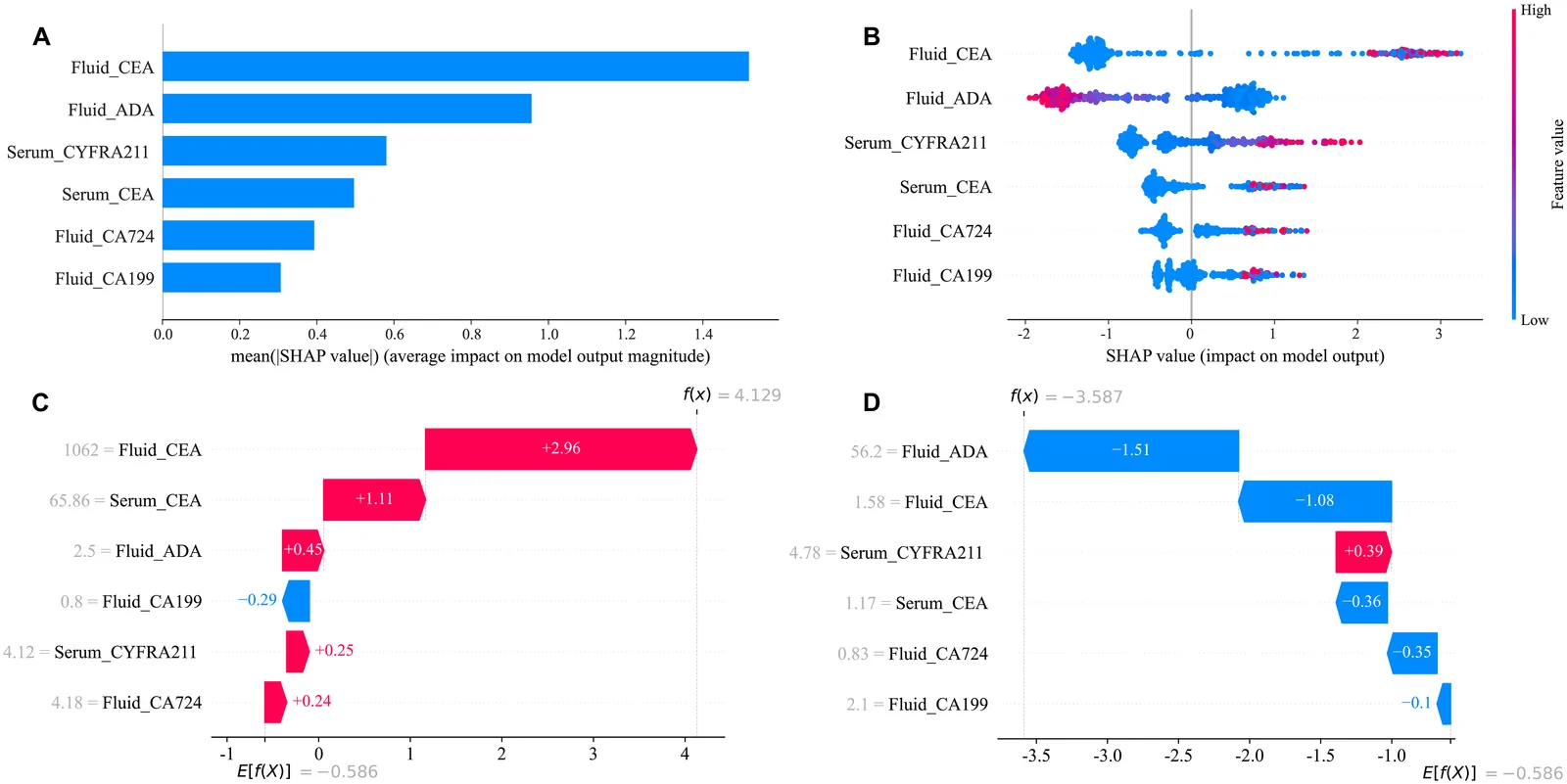
SHAP-based interpretability visualization for the XGBoost model. **(A)** Feature importance ranking by mean absolute SHAP value, indicating each feature’s average contribution to the model predictions. **(B)** SHAP beeswarm plot showing the distributions of SHAP values across samples, with color gradient representing the magnitude of the original feature values (red = high, blue = low). **(C, D)** SHAP waterfall plot demonstrating individual feature contributions for a representative patient case.

**Figure 5:**
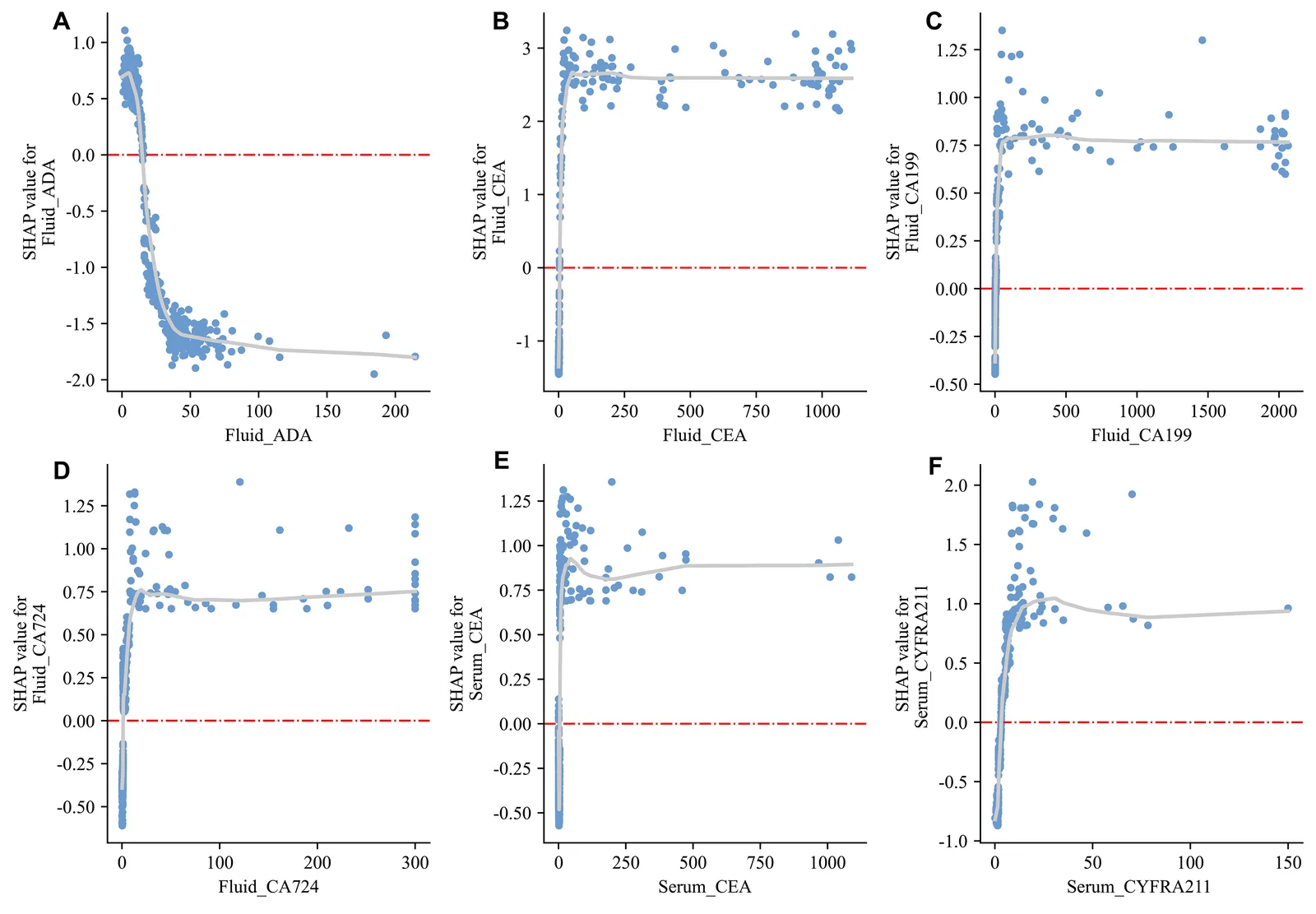
SHAP dependency plots with LOWESS regression curves for six key features in the XGBoost model. **(A-F)** association between the actual values of the selected variables and their corresponding SHAP values. Positive SHAP values indicates a contribution toward prediction MPE group, whereas negative SHAP values reflects a tendency toward prediction BPE group.

SHAP plots were used to visualize the impact of predictive variables on the XGBoost model output and to illustrate the contribution of multiple factors. As shown in **Figure 4A**, fluid CEA and fluid ADA were the most influential variables for prediction probability of MPE. The SHAP beeswarm plot shows the distribution and direction of each feature’s contribution to the model output. Higher pleural CEA, lower pleural ADA, higher serum CYFRA211, higher serum CEA, higher fluid CA724 and higher fluid CA199 were associated with higher predicted probability of MPE (**Figure 4B**). Finally, SHAP waterfall plots were used to verify the interpretability for two representative cases (**Figures 4C, D**). SHAP dependence plots were employed to visualize nonlinear relationships between individual features and model predictions (**Figures 5A–F**). These plots clearly show the directional effects and threshold patterns of key variables on model predictions, improving interpretability and clinical utility.

## Discussion

In this study, we constructed and compared six ML methods to distinguish MPE from BPE based on clinical and laboratory data. Our results showed that the XGBoost model exhibited the best performances for diagnosing MPE, with an AUC of 0.988 (95% CI: 0.982-0.993), the lowest brier score 0.037 (95% CI: 0.029-0.045), and high values for accuracy of 0.951, sensitivity of 0.891, specificity of 0.984, and F1 score of 0.928 in the training set. The consistent results in the test and external validation sets further indicated high model robustness. Besides, the XGBoost model also indicated higher net benefits in the test and external validation sets. Additionally, SHAP analysis showed that fluid CEA and fluid ADA were the most influential variables for prediction probability of MPE. We utilized routinely detected laboratory indicators to develop a model with high diagnostic performance, which might be well applied in clinical practice. This method is an effective, less invasive and low-cost auxiliary diagnostic method for MPE. Therefore, the results of our study may be helpful for clinicians to make decisions.

Recently, ML have demonstrated high AUC and accuracy in the diagnosis and differential diagnosis of MPE (17, 22). However, previous studies have limitations in terms of feature selection or model evaluation. A study of Wei et al. suggested that the XGBoost model outperformed in distinguishing MPE from BPE than LR, RF, SVM, and MLP (23). The diagnostic AUC of XGBoost was 0.918. However, the lack of feature selection method led to the inclusion of non-specific features, such as calcium (23). Besides, the fluid CYFRA21–1 and fluid NSE were not included in Wei’s study. Importantly, the results of Wei’s study were not validated by independent external data. Additionally, Kim et al. developed five ML models to classify pleural effusions into five categories of PEs in a large cohort of 2,253 PE patients, including transudative, malignant, parapneumonic, tuberculous, and other (24). The results showed that the LightGBM outperformed the other four models for differential diagnosis of the common causes of PEs using the selected 18 features (24). However, incorporating 18 features into the model does indeed improve the diagnostic AUC for MPE. However, too many indicators extremely make LightGBM model cumbersome and limits its clinical application. Zhang et al. focus on the combination ML method and tumor markers in identifying MPE from non-MPE (17). The authors found that the AUC of the XGBoost model combining CEA and carbohydrate antigen 153 reached 0.921 (17). However, the sample size of this study is relatively small (only 319 cases), and the tumor indicators in the PE were not taken into consideration.

Compared with aforementioned studies, our model incorporates indicators from both peripheral blood and PE, thereby further enhancing its applicability. In addition, we employed a feature selection strategy by intersecting results from REF and Boruta to ensure the clinical interpretability of the optimal model. This selection approach not only mitigated collinearity and overfitting, but also improve the interpretability and generalizability of the model. However, common limitations across these studies include dataset quality (such as imbalance and sparsity), feature selection, model overfitting, and generalization issues (21). Future studies should focus on expanding and diversifying datasets, including laboratory and imaging data, potentially through automated data collection methods to enhance model performance. The interpretability of ML models is also a challenge. Although ML models often exhibit high AUC and accuracy, their complexity limits their practical application. In this context, the SHAP might be employed to gain insights into the explainability of ML models (25).

Considering the black-box nature of the ML model, the SHAP method was used to quantify the contribution of each feature to the prediction model (25). SHAP is a unified measure of feature importance for ML models. A SHAP value summary plot visualizes the overall impact of each feature on the model’s output. Features with a larger magnitude of SHAP value have a greater impact on the model’s output. In this study, fluid CEA and fluid ADA were the two most important features. Using the SHAP summary plot, higher fluid CEA values were associated with a greater positive contribution to the model output. However, the fluid ADA had the negative contributions to the model output. The results are consistent with the widespread use of these two indicators in MPE and TPE diagnosis. Notably, several studies have reported the potential diagnostic value of fluid CEA and serum CEA in MPE (26, 27). Although fluid CEA has high specificity, the diagnostic sensitivity was relatively lower (26, 27). Besides, the lack of cutoff values, different detection methods, and sample size have led to inconsistent or contradictory results of fluid CEA. Therefore, the fluid CEA level has a relatively high specificity (more than 90%) for diagnosing MPE, but its sensitivity varies (35% to 89%). Additionally, the fluid CEA level in malignant mesothelioma was rarely elevated (28). ADA, an enzyme involved in purine metabolism, has emerged as a biologically relevant marker associated with tuberculosis infection. ADA plays an important role in cellular immunity by regulating the proliferation and differentiation of T-lymphocytes which are essential components of the host immune response against *M. tuberculosis* (29). Therefore, elevated ADA levels are associated with TPE, not MPE. Hence, SHAP analysis significantly enhances the understanding and trust for clinicians in the diagnostic model and facilitates its practical application. Therefore, the use of ML for the differential diagnoses of MPE is useful for clinicians, in that ML incorporates multiple factors, such as patient demographics, and the variables in blood and PE. ML could help clinicians distinguish MPE patients from BPE, which is clearly superior to a single indicator, such as fluid CEA.

Several limitations should be noted in the study. First, the study was a retrospective cohort study, which might suffer from inherent biases. Although we validated our results with an external cohort, the external sample size was small. Second, the present study only included PE patients from one geographic region (Ningbo) of China, and excluded undiagnosed PE patients, which could limit the generalizability of predictive models and require validation from distinct geographic regions in China. Finally, only clinical and laboratory variables were incorporated in the study. Due to the long-time span of the patient data (from 2016 to 2025), the imaging CT reports of several patients from the early stage are no longer available. Therefore, we did not include CT-related examinations for analysis. Clinical, laboratory and imaging variables can be integrated to construct a new diagnostic ML model of MPE in the future. Therefore, further prospective studies from several medical centers are necessary to validate the generalizability and clinical practicability of our results.

## Conclusions

In summary, we developed and validated a six-feature XGBoost model to distinguish MPE from BPE. The XGBoost model exhibited better AUC, accuracy, sensitivity, and specificity in identifying MPE. The model, by incorporating available laboratory variables, will be more conducive to its clinical application. Moreover, SHAP analysis further strengthened the interpretability for the model and key features. The generalizability and practicability of our results required validation by prospective, multicenter, and regional studies in the future.

## Data Availability

The original contributions presented in the study are included in the article/**Supplementary Material**. Further inquiries can be directed to the corresponding author.

## Supplementary material

Supplementary Figure 1 The Spearman correlation matrix among candidate features was presented in the training cohort, with statistical significance indicated by asterisks (**p* < 0.05, ***p* < 0.01, ****p* < 0.001). **(A, B)** Spearman correlation heatmaps for patients diagnosed with MPE and BPE.

Supplementary Figure 2 Precision-recall (PR) curves showing the trade-off between precision and recall for the XGB model in the training, test and the external validation sets.
