# Supplementary material for: Development and external validation of machine-learning models for diagnosing malignant pleural effusion
Source: Front Oncol. 2026 Aug 17;16:1880122. doi: 10.3389/fonc.2026.1880122 (PMC13526590; doi:10.3389/fonc.2026.1880122)
Supplement: Supplementary file 1 [file Table1.docx]

Supplementary Table 1 Diagnostic criteria for malignant pleural effusion and benign pleural effusion

| Disease type | Diagnostic criteria |
| --- | --- |
| Malignant pleural effusion | (1) Malignant cells were detected in the PE with cytological smears, cell blocks, or pleural biopsy;  (2) Imaging showed malignant pleural nodules or thickening with a confirmed extrapleural primary malignancy; |
| Tuberculous pleural effusion | (1) Positive Acid-fast stains of pleural fluid smears or pleural biopsy;  (2) Positive *Mycobacterium tuberculosis* culture of pleural effusion or pleural biopsy tissue;  (3) Positive Xpert MTB/RIF test or PCR of pleural effusion or pleural biopsy tissue;  (4) Both presence of granulomatous inflammation in the pleura biopsy  by histologic examination and clinical response to anti-TB treatment |
| Parapneumonic effusion | The majority of pleural effusions are secondary to bacterial pneumonia and was resolved after antibiotic treatment. |
| Cardiac-related pleural effusion | 2016 ESC Guidelines for the diagnosis and treatment of acute and chronic heart failure. |
| Rheumatoid arthritis | The National Institute for Health and Care Excellence guideline for the diagnosis and management of rheumatoid arthritis in adults. |
