# Supplementary material for: Development and external validation of machine-learning models for diagnosing malignant pleural effusion
Source: Front Oncol. 2026 Aug 17;16:1880122. doi: 10.3389/fonc.2026.1880122 (PMC13526590; doi:10.3389/fonc.2026.1880122)
Supplement: Supplementary file 2 [file Table2.docx]

Supplementary Table 2 The collected indexes in the Materials and Methods

| Demographics | Serum indexes | Fluid indexes |
| --- | --- | --- |
| Age (years) | CRP (mg/L) | WBC (×10^8^/L) |
| Sex (Female/Male) | ESR (mm/h) | Neutrophil (×10^8^/L) |
| Smoking history | LDH (U/L) | Lymphocyte (×10^8^/L) |
|  | CEA (ng/mL) | TP (g/L) |
|  | CA125 (U/mL) | Glucose (mmol/L) |
|  | CA19-9 (U/mL) | ADA (U/L) |
|  | CYFRA21-1 (ng/mL) | LDH (U/L) |
|  | NSE (ng/mL) | CEA (ng/mL) |
|  | CA724 (U/mL) | CA125 (U/mL) |
|  |  | CA19-9 (U/mL) |
|  |  | CYFRA21-1 (ng/mL) |
|  |  | NSE (ng/mL) |
|  |  | CA724 (U/mL) |

Abbreviations: WBC, white blood cell; TP, total protein; ADA, adenosine deaminase; LDH, lactate dehydrogenase; CEA, carcinoembryonic antigen; CA125, carbohydrate antigen 125; CA19-9, carbohydrate antigen 19-9; CYFRA21-1, cytokeratin 19 fragment; NSE, neuron-specific enolase; CA724, carbohydrate antigen 724; CRP, high-sensitivity C-reactive protein; ESR, erythrocyte sedimentation rate
