# Supplementary material for: Development and external validation of machine-learning models for diagnosing malignant pleural effusion
Source: Front Oncol. 2026 Aug 17;16:1880122. doi: 10.3389/fonc.2026.1880122 (PMC13526590; doi:10.3389/fonc.2026.1880122)
Supplement: Supplementary file 3 [file Table3.docx]

Supplementary Table 3 The origins of pleural effusion from the two hospitals

| Disease causes | First Affiliated Hospital (n, %) | Affiliated People Hospital (n, %) |
| --- | --- | --- |
| MPE | 550 | 115 |
| Lung cancer | 393 (72%) | 97 (84%) |
| Breast cancer | 27 (5%) | 5 (4%) |
| Ovarian cancer | 13 (2%) | 2 (2%) |
| Lymphoma | 38 (7%) | 3 (3%) |
| Mesothelioma | 16 (3%) | 2 (2%) |
| Other cancers | 63 (11%) | 6 (5%) |
| BPE | 980 | 129 |
| TPE | 449 (46%) | 67 (52%) |
| PPE | 205 (21%) | 20 (16%) |
| Empyema | 103 (10%) | 11 (8%) |
| Heart failure | 146 (15%) | 14 (11%) |
| Other diseases | 77 (8 %) | 17 (13%) |

Abbreviations: MPE, malignant pleural effusion; BPE, benign pleural effusion; TPE, tuberculous pleural effusion; PPE, parapneumonic pleural effusion.
