# Supplementary material for: Development and external validation of machine-learning models for diagnosing malignant pleural effusion
Source: Front Oncol. 2026 Aug 17;16:1880122. doi: 10.3389/fonc.2026.1880122 (PMC13526590; doi:10.3389/fonc.2026.1880122)
Supplement: Supplementary file 4 [file Table4.docx]

Supplementary Table 4 Employing recursive feature elimination method and 5 fold cross-validation for machine learning feature screening.

| Features | Importance | Mean ROC | Fold 1 ROC | Fold 2  ROC | Fold 3  ROC | Fold 4  ROC | Fold 5  ROC |
| --- | --- | --- | --- | --- | --- | --- | --- |
| **Fluid CEA** | **0.429** | 0.905 | 0.928 | 0.922 | 0.913 | 0.892 | **0.869** |
| **Serum CEA** | **0.188** | 0.928 | 0.949 | 0.948 | 0.940 | 0.891 | **0.911** |
| **Serum CYFRA211** | **0.108** | 0.955 | 0.97 | 0.956 | 0.963 | 0.944 | **0.943** |
| **Fluid CA724** | **0.064** | 0.955 | 0.968 | 0.949 | 0.960 | 0.948 | **0.949** |
| **Fluid CA19-9** | **0.029** | 0.959 | 0.969 | 0.955 | 0.963 | 0.946 | **0.962** |
| **Fluid ADA** | **0.033** | 0.977 | 0.986 | 0.974 | 0.987 | 0.968 | **0.972** |
| Fluid NSE | 0.022 | 0.978 | 0.983 | 0.979 | 0.991 | 0.962 | 0.975 |
| Serum NSE | 0.019 | 0.980 | 0.982 | 0.977 | 0.991 | 0.972 | 0.976 |
| Fluid CYFRA211 | 0.012 | 0.977 | 0.980 | 0.978 | 0.988 | 0.962 | 0.977 |
| Fluid total protein | 0.012 | 0.979 | 0.976 | 0.982 | 0.99 | 0.972 | 0.976 |
| Fluid CA125 | 0.01 | 0.980 | 0.983 | 0.977 | 0.991 | 0.969 | 0.978 |
| Serum CA19-9 | 0.0085 | 0.980 | 0.980 | 0.981 | 0.991 | 0.975 | 0.97 |
| Serum CRP | 0.0098 | 0.980 | 0.986 | 0.982 | 0.990 | 0.969 | 0.971 |
| Fluid WBC | 0.0063 | 0.979 | 0.979 | 0.983 | 0.991 | 0.970 | 0.973 |
| Age | 0.0086 | 0.980 | 0.982 | 0.983 | 0.992 | 0.970 | 0.973 |
| Fluid LDH | 0.007 | 0.980 | 0.979 | 0.983 | 0.993 | 0.973 | 0.973 |
| Fluid lymphocyte | 0.0055 | 0.981 | 0.984 | 0.98 | 0.991 | 0.971 | 0.981 |
| Fluid GLU | 0.0062 | 0.980 | 0.986 | 0.984 | 0.992 | 0.967 | 0.973 |
| Serum CA724 | 0.0038 | 0.977 | 0.976 | 0.982 | 0.990 | 0.965 | 0.97 |
| Fluid neutrophil | 0.0043 | 0.978 | 0.981 | 0.979 | 0.990 | 0.971 | 0.971 |
| Serum LDH | 0.0043 | 0.979 | 0.982 | 0.981 | 0.991 | 0.968 | 0.973 |
| Serum CA125 | 0.0041 | 0.981 | 0.984 | 0.98 | 0.992 | 0.975 | 0.973 |
| ESR | 0.0036 | 0.979 | 0.980 | 0.982 | 0.990 | 0.971 | 0.973 |
| Smoking history | 0.0009 | 0.979 | 0.975 | 0.985 | 0.991 | 0.968 | 0.974 |
| Sex | 0.0002 | 0.979 | 0.983 | 0.979 | 0.989 | 0.97 | 0.975 |

Abbreviations: ROC, receiver operating characteristic; WBC, white blood cell; CEA, carcinoembryonic antigen; ADA, adenosine deaminase; LDH, lactate dehydrogenase; CA19-9, carbohydrate antigen 19-9; CYFRA21-1, cytokeratin 19 fragment; CA724, carbohydrate antigen 724; NSE, neuron-specific enolase; CA125, carbohydrate antigen 125; CRP, high-sensitivity C-reactive protein; GLU, Glucose; ESR, erythrocyte sedimentation rate.
