# Supplementary material for: Development and external validation of machine-learning models for diagnosing malignant pleural effusion
Source: Front Oncol. 2026 Aug 17;16:1880122. doi: 10.3389/fonc.2026.1880122 (PMC13526590; doi:10.3389/fonc.2026.1880122)
Supplement: Supplementary file 5 [file Table5.docx]

Supplementary Table 5 Collinearity Analysis of Candidate and Selected Variables.

| Variable | VIF (All candidate variables) | VIF (Selected features) | Inclusion in final model |
| --- | --- | --- | --- |
| Fluid WBC | 7.30 |  |  |
| Fluid Neutrophil | 6.65 |  |  |
| **Fluid CEA** | 2.26 | 1.94 | **Yes** |
| **Fluid ADA** | 1.99 | 1.05 | **Yes** |
| Fluid LDH | 1.94 |  |  |
| Fluid Lymphocyte | 1.86 |  |  |
| **Fluid CA19-9** | 1.73 | 1.28 | **Yes** |
| Fluid CYFRA211 | 1.71 |  |  |
| **Serum CEA** | 1.61 | 1.49 | **Yes** |
| **Fluid CA724** | 1.54 | 1.32 | **Yes** |
| Sex | 1.54 |  |  |
| Fluid NSE | 1.52 |  |  |
| Serum CA19-9 | 1.52 |  |  |
| Fluid CA125 | 1.52 |  |  |
| Smoking history | 1.49 |  |  |
| Serum CRP | 1.43 |  |  |
| Age | 1.43 |  |  |
| Serum CA125 | 1.42 |  |  |
| Serum CA724 | 1.41 |  |  |
| Fluid GLU | 1.40 |  |  |
| Fluid Total protein | 1.38 |  |  |
| **Serum CYFRA211** | 1.32 | 1.09 | **Yes** |
| Serum NSE | 1.31 |  |  |
| ESR | 1.28 |  |  |
| Serum LDH | 1.18 |  |  |

Abbreviations: WBC, white blood cell; CEA, carcinoembryonic antigen; ADA, adenosine deaminase; LDH, lactate dehydrogenase; CA19-9, carbohydrate antigen 19-9; CYFRA21-1, cytokeratin 19 fragment; CA724, carbohydrate antigen 724; NSE, neuron-specific enolase; CA125, carbohydrate antigen 125; CRP, high-sensitivity C-reactive protein; GLU, Glucose; ESR, erythrocyte sedimentation rate.

Note: Variance inflation factors (VIFs) were calculated for all candidate variables, and only those with VIF < 5 were ultimately selected for the final model (marked as “Yes”).
