# Supplementary material for: Development and external validation of machine-learning models for diagnosing malignant pleural effusion
Source: Front Oncol. 2026 Aug 17;16:1880122. doi: 10.3389/fonc.2026.1880122 (PMC13526590; doi:10.3389/fonc.2026.1880122)
Supplement: Supplementary file 6 [file Table6.docx]

Supplementary Method Hyperparameter tuning strategy, related to Method Details

1. Performing grid search for LR

Fitting 5 folds

Best parameters for log_reg: {'C': 0.01, 'max_iter': 2000, 'penalty': 'l2', 'solver': 'lbfgs'}

2. Performing grid search for RF

Fitting 5 folds

Best parameters for rf: {'bootstrap': True, 'max_depth': None, 'max_features': 'sqrt', 'max_samples': 0.8, 'min_samples_leaf': 5, 'min_samples_split': 10, 'n_estimators': 500, 'oob_score': True}

3. Performing grid search for SVM

Fitting 5 folds

Best parameters for svm: {'C': 0.01, 'gamma': 'scale', 'kernel': 'linear'}

4. Performing grid search for XGB

Fitting 5 folds

Best parameters for xgb: {'colsample_bytree': 0.7, 'gamma': 0.3, 'learning_rate': 0.05, 'max_depth': 3, 'min_child_weight': 3, 'n_estimators': 100, 'reg_alpha': 0.1, 'reg_lambda': 1, 'subsample': 0.8}

5. Performing grid search for LGB

Fitting 5 folds

Best parameters for lgb: {'boosting_type': 'gbdt', 'feature_fraction': 0.6, 'learning_rate': 0.03, 'max_depth': 3, 'min_child_samples': 20, 'n_estimators': 100, 'num_leaves': 15, 'reg_alpha': 1, 'reg_lambda': 5, 'subsample': 0.6}

6. Performing grid search for MLP

Fitting 5 folds

Best parameters for mlp: {'activation': 'relu', 'alpha': 0.001, 'early_stopping': True, 'hidden_layer_sizes': (50,), 'learning_rate_init': 0.001, 'max_iter': 500, 'solver': 'adam', 'validation_fraction': 0.2}
